# Experimental design, formulation and *in vivo* evaluation of a novel topical *in situ* gel system to treat ocular infections

**DOI:** 10.1371/journal.pone.0248857

**Published:** 2021-03-19

**Authors:** Anroop B. Nair, Jigar Shah, Shery Jacob, Bandar E. Al-Dhubiab, Nagaraja Sreeharsha, Mohamed A. Morsy, Sumeet Gupta, Mahesh Attimarad, Pottathil Shinu, Katharigatta N. Venugopala

**Affiliations:** 1 Department of Pharmaceutical Sciences, College of Clinical Pharmacy, King Faisal University, Al-Ahsa, Saudi Arabia; 2 Department of Pharmaceutics, Institute of Pharmacy, Nirma University, Ahmedabad, Gujarat, India; 3 Department of Pharmaceutical Sciences, College of Pharmacy, Gulf Medical University, Ajman, United Arab Emirates; 4 Department of Pharmaceutics, Vidya Siri College of Pharmacy, Bangalore, India; 5 Faculty of Medicine, Department of Pharmacology, Minia University, El-Minia, Egypt; 6 Department of Pharmacology, M. M. College of Pharmacy, Maharishi Markandeshwar (Deemed to be University), Mullana, India; 7 Department of Biomedical Sciences, College of Clinical Pharmacy, King Faisal University, Al-Ahsa, Saudi Arabia; 8 Department of Biotechnology and Food Technology, Durban University of Technology, Durban, South Africa; Bhagwan Mahvir College of Pharmacy, INDIA

## Abstract

*In situ gels* have been extensively explored as ocular drug delivery system to enhance bioavailability and efficacy. The objective of present study was to design, formulate and evaluate ion-activated *in situ* gel to enhance the ocular penetration and therapeutic performance of moxifloxacin in ophthalmic delivery. A simplex lattice design was utilized to examine the effect of various factors on experimental outcomes of the *in situ* gel system. The influence of polymers (independent variables) such as gellan gum (X_1_), sodium alginate (X_2_), and HPMC (X_3_) on gel strength, adhesive force, viscosity and drug release after 10 h (Q_10_) were assessed. Selected formulation (MH7) was studied for *ex vivo* permeation, *in vivo* irritation and pharmacokinetics in rabbits. Data revealed that increase in concentration of polymers led to higher gel strength, adhesive force and viscosity, however, decreases the drug release. MH7 exhibited all physicochemical properties within acceptable limits and was stable for 6 months. Release profile of moxifloxacin from MH7 was comparable to the check point batches and followed Korsmeyer-Peppas matrix diffusion-controlled mechanism. Ocular irritation study signifies that selected formulation is safe and non-irritant for ophthalmic administration. *In vivo* pharmacokinetics data indicates significant improvement of moxifloxacin bioavailability (*p <* 0.0001) from MH7, as evidenced by higher C_max_ (727 ± 56 ng/ml) and greater AUC (2881 ± 108 ng h/ml), when compared with commercial eye drops (C_max_; 503 ± 85 ng/ml and AUC; 978 ± 86 ng h/ml). In conclusion, developed *in situ* gel system (MH7) could offers a more effective and extended ophthalmic therapy of moxifloxacin in ocular infections when compared to conventional eye drops.

## Introduction

A key challenge frequently encountered during the development of ophthalmic delivery systems is the achievement of desired drug level at the target site, particularly within the anterior cavity of the eye, for sufficient time. This is mainly due to the complex anatomy and highly selective corneal barriers, which limit the entry of any exogenous substances to the ocular tissues [1,2]. Different types of ophthalmic vehicles like eye drops, ointments, gels and polymeric ocular inserts were developed in an attempt to enhance the pre-corneal residence time [3]. Among the various ophthalmic dosage forms evaluated so far, *in situ* gel drug delivery systems has been an extensive area of research during last few decades. *In situ* gels are attractive since it can be suitably applied as drops or solutions into the conjunctival sac, wherein they undergo a phase conversion into a gel state upon exposure to either pH of the tear fluid, ocular surface temperature, or ions exists on the tear film [4,5]. Transition to gel state in the corneal surface extends the ocular residence resulting in better ocular bioavailability by minimizing rapid pre-corneal elimination, particularly due to nasolacrimal drainage and eye blinking [6]. It can also reduce the poor compliance due to frequent administration and risk of undesirable side effects associated with systemic drug absorption by virtue of pre-corneal elimination [7].

*In situ* activated transparent gel formulations is ideal for ocular therapy as it can be administered as liquid dosage form and avoids blurred vision. Besides, they exhibit excellent physicochemical characteristics such as bioadhesion, ocular tolerance and sustained drug release properties than conventional ophthalmic preparations as a consequence of prolonged pre-corneal residence time. Presently, these type of dosage forms is employed in many ocular conditions such as glaucoma, dry eye syndrome, Sjogren’s syndrome, age related macular degeneration and trachoma [8]. Recently, low acyl gellan gum has received much attention as a gelling agent in various drug delivery systems because of its excellent mechanical properties and favorable rheological behavior [9–11]. The potential of gellan gum as an ophthalmic vehicle was also demonstrated in various studies [12,13].

Moxifloxacin, a fourth-generation broad-spectrum fluoroquinolone derivative has exceptional activity against various gram-negative and gram-positive pathogens. It is used topically for treating various ocular infections including conjunctivitis, bacterial keratitis, and keratoconjunctivitis. This drug exists as unionized form at neutral pH of tears and thereby causing an enhanced corneal permeation and 2–3 folds higher concentration in the aqueous humour than other fluoroquinolones derivatives [14]. Few attempts have been made to enhance the ocular delivery of moxifloxacin using carriers including liposomes, microemulsions and nanoemulsions [15–18]. In sight of this, the objective of present study was to demonstrate the potential of optimized *in situ* ion activated gelling system to improve moxifloxacin therapeutic efficacy in ocular therapy. Simplex lattice design plan was constructed to optimize and evaluate *in situ* ophthalmic gel comprised of moxifloxacin (0.5% w/v). Various gelling agents and viscosity enhancers were evaluated to assess their potential for developing *in situ* gel. Formulation parameters like gelation time, viscoelastic nature, adhesive force and release behaviour of the *in situ* gelling system were optimized. *Ex vivo* as well as *in vivo* evaluation of optimized *in situ* gel (MH7) have been carried out to confirm the sustained ophthalmic delivery and treatment efficacy of moxifloxacin.

## Materials and methods

### Chemicals

Moxifloxacin hydrochloride (purity of 99.99%), Poloxamer 188 and Poloxamer 407 were provided by Zydus Cadila Ltd., Ahmedabad, India. Sodium alginate (Zydus Cadila, Bangalore, India) and carbopol 940P (Torrent Pharma, Ahmedabad, India) were received as gratis sample. Kelcogel F (low acyl gellan gum) was donated by CPKelco, Surrey, UK. Hydroxypropyl methylcellulose (HPMC) F4M, and methyl cellulose (MC) were obtained from Colorcon Limited, Dartford, England. Calcium chloride, mannitol and methyl paraben were purchased commercially from CDH Ltd., Mumbai, India.

### Drug analysis

Quantification of moxifloxacin was performed using high-performance liquid chromatography (HPLC) system (PU 2080, UV– 2075 plus, Jasco, Tokyo, Japan). The HPLC system utilized is made of Phenomenex C-18 column (150 × 4.6 mm, i.d 5 μm) connected to UV-Visible detector (MD-4010) and a software for data acquisition (ChromNAV 2.0, Jasco, Tokyo, Japan). Chromatographic separation of moxifloxacin was accomplished using a mixture of mobile phase consist of acetonitrile: potassium dihydrogen ortho-phosphate (0.02 mM) 20:80 v/v, adjusted to a pH 4.5 with phosphoric acid [19]. The temperature in the C-18 column was set at 25°C, while the rate of solvent flow was fixed at 1 ml/min to elute moxifloxacin and was detected at 305 nm. Regression analysis indicates good linearity when moxifloxacin concentration was in the range of 25–300 ng/ml (r^2^ = 0.995).

### Formulation of *in situ* gels

Initial trials were performed to identify the most appropriate polymer system (s) at suitable proportion, which is efficient in forming *in situ* ophthalmic gels having desirable physical properties. The sol to gel phase transition potential of *in situ* gel formulations prepared by ion activation (gellan gum and sodium alginate), pH triggering (carbopol 940P) and temperature (poloxamer 188/407) were assessed. HPMC or MC was used as viscosity enhancing agents. Based on literature search, the concentrations of polymers were selected for the different types of *in situ* gel formulations. The composition of different formulation batches (M1-M14) are given in Table 1.

**Table 1:** Composition of prepared *in situ* gel formulations.

Ingredients	Batch code													
	M1	M2	M3	M4	M5	M6	M7	M8	M9	M10	M11	M12	M13	M14
**Gellan gum (% w/v)**	0.5	0.7	-	-	0.5	0.7	0.5	0.7	-	-	-	-	-	-
**Sodium alginate (% w/v)**	-	-	0.3	0.5	0.3	0.3	0.3	0.3	-	-	-	-	-	-
**Poloxamer 188 (% w/v)**	-	-	-	-	-	-	-	-	18	25	-	-	-	-
**Poloxamer 407 (% w/v)**	-	-	-	-	-	-	-	-	-	-	18	25	-	-
**Carbopol 940P (% w/v)**	-	-	-	-	-	-	-	-	-	-	-	-	0.2	0.4
**HPMC (% w/v)**	-	-	-	-	-	-	0.4	0.4	0.5	-	0.5	-	0.5	-
**Methyl cellulose (% w/v)**	-	-	-	-	0.5	0.5	-	-	-	0.5	-	0.5	-	0.5
**Phosphate buffer pH 7.4 (ml)**	100	100	100	100	100	100	100	100	-	-	-	-	-	-
**Acetate buffer pH 6.5 (ml)**	-	-	-	-	-	-	-	-	100	100	100	100	-	-
**Boric acid buffer pH 4.7 (ml)**	-	-	-	-	-	-	-	-	-	-	-	-	100	100

Amount of moxifloxacin incorporated in all formulations is 0.5% w/v. HPMC–Methocel F4M Premium USP.

### Gellan gum based *in situ* gel

Aqueous solutions of *in situ* gel systems (M1-M8) were formulated by dissolving polymers (gellan gum, sodium alginate, HPMC or MC) in warm (70°C) phosphate buffer (pH 7.4) by constant stirring [20]. The polymeric solution was subsequently cooled to room temperature (25 ± 1°C) to which specified quantities of moxifloxacin (0.5% w/v) was added and stirred until completely dissolved. Terminal sterilization of the ophthalmic gels was carried out by heating in an autoclave and kept in a refrigerator until further investigation.

### Poloxamer based *in situ* gel

Poloxamer *in situ* forming gels (M9-M12) were formulated by the modified cold method. Briefly, poloxamer (P188 and P407), HPMC and MC was slowly added to the required amount of cold acetate buffer (pH 6.5) containing moxifloxacin (0.5% w/v) with constant stirring. The partially dissolved Poloxamer solution was stored in refrigerator and stirred occasionally until a clear homogenous solution is obtained.

### Carbopol based *in situ* gel

Gels (M13 and M14) were formulated by dissolving carbopol 940P in a half quantity of modified boric acid buffer (pH 4.7) with constant stirring. HPMC and MC were dissolved in a separate container using remaining half of vehicle with slight heating. The two solutions were then mixed and the moxifloxacin (0.5% w/v) was added. The solutions were then equilibrated at room temperature (25 ± 1°C) for 24 h.

### Evaluation of gels

#### Appearance and pH

Clarity testing was performed on all developed formulations by visual observation of the samples to examine the presence of any transparent or coloured particulate matters or turbidity. The pH of the various gels was determined by calibrated pH meter (Mettler Toledo MP-220, Greifensee, Switzerland) at 25 ± 0.5°C as per the standard procedure.

### Drug content

The polypropylene vials containing the accurately weighed quantity (1 g) of formulations were mixed with mobile phase using a laboratory shaker (EIE 405, EIE Instruments, Ahmedabad, India) for 2–3 min. Lastly, aliquot of the solution was filtered through a filter membrane (pore size of 0.2 μm), diluted using simulated tear fluid (STF) and injected into the HPLC system.

### *In vitro* gelation

The gelation of gels was assessed using a polypropylene vial having STF as gelation solution, equilibrated at 34 ± 0.5°C using a water bath. Aliquot of each preparation (100 μl) were precisely transferred into separate vial followed by the gradual addition of STF (2 ml) using a micropipette. Gelling capacity was observed by visual monitoring of the formation and measuring the time needed for gelation and the time required to dissolve the gel formed [21].

### Rheology

The viscosity of *in situ* gels was determined at different angular velocity (0.5 to 100 rpm) at 34 ± 1°C using a Brookfield Viscometer (LVDVI prime, Middleborough, MA, USA). A typical run involved consecutively varying and reversing the angular velocity for an identical period of 6 sec at a controlled ramp speed [22]. To assess the variation of rheology typically observed after ocular application, viscosity of gels was also determined after thinning the gel with STF in 25:7 ratios.

### Gel strength and adhesive force

Gel strength and adhesive force was measured utilizing a QTS Texture analyzer (Brookfield Engineering Labs, Inc., Middleboro, MA, USA) (S1 Fig). Preparation with STF were placed into a cylindrical holder while precautions was taken to prevent the air entrapment in samples. A cylindrical probe with approximately 38 mm of diameter was allowed to enter into sample gel at a rate of 1 mm/s into a depth of 10 mm at a measurable force. The gel strength (as peak load) and adhesive force (work needed to disturb the attractive forces between cylindrical probe and sample) was computed from the resulting load–time plots [23,24].

### Experimental design

Experimental design to optimize the ion activated *in situ* gel contain moxifloxacin was carried out using simplex lattice DoE. The statistical technique was constructed to investigate the influence of independent variables like quantity of polymers [gellan gum (X_1_), sodium alginate (X_2_), and HPMC (X_3_)] on dependent variables such as gel strength (G), adhesive force (A), rheological property as viscosity (v) and moxifloxacin release after 10 h (Q_10_). The DoE outline as well as responses of MH1-MH7 gels of the design were presented in Table 2.

**Table 2:** Composition of experimental design batches.

Variable level in coded form					
Batch code	Transformed fractions				
	X_1_	X_2_		X_3_	
MH1	1	0		0	
MH2	0	1		0	
MH3	0	0		1	
MH4	0.5	0.5		0	
MH5	0.5	0		0.5	
MH6	0	0.5		0.5	
MH7	0.33	0.33		0.33	
**Independent variables**	**Coded values**	**Actual values**			
X_1_ = Amount of gellan gum (%)		X_1_	X_2_		X_3_
X_2_ = Amount of sodium alginate (%)	0	0.4	0.2		0.3
X_3_ = Amount of HPMC (%)	1	0.6	0.4		0.5

### Validation of applied design

Design layout was practically evaluated by a checkpoint batch. For the proposed in situ gelling system, parameters like gelling strength, viscosity, bioadhesion and *in vitro* drug release are important and were selected as various criteria for check point analysis. Based on preliminary studies and to validate the experimental design model, two extra checkpoint batches were prepared. Briefly, the transformed proportions of the coded values were inserted into the corresponding polynomial response equation to find the theoretical response. Then actual response was practically done for the checkpoint batch and correlated with the theoretical response. The validation of the chosen design was demonstrated by the close values of the results [25].

### *In vitro* release

A Franz diffusion cell (Logan Instruments Ltd., Somerset, NJ) having an exposed surface area of 0.79 cm^2^ and STF (pH 7.4) as the dissolution medium was utilized for carrying out *in vitro* release of moxifloxacin from prepared gels [26]. Prepared formulations (MH1-MH7) or check point batches (MH8 and MH9) or control was placed on a cellophane dialysis film (MWCO 12–14 kDa) previously fixed between the upper and lower chambers. The temperature of the assembly was fixed at 37 ± 0.5°C and receiver solution was mixed at 50 rpm using a magnetic stirrer. At suitable time intervals, aliquots of sample (1 ml) were drawn and substituted with the equivalent amount of STF held at the same temperature. A control experiment was performed at same experimental conditions using similar strength of moxifloxacin solution. The samples were later diluted with mobile phase and quantified for moxifloxacin by HPLC. The data collected were analyzed to calculate regression coefficient (r^2^) and interpret release kinetics using various mathematical models [27].

### *Ex vivo* permeation

Trans-corneal permeation of moxifloxacin from optimized formulation (MH7) and control (commercial eye drops—Vigamox^TM^; equivalent to 5 mg of moxifloxacin per ml) was carried out using goat cornea as membrane. Whole eyeballs of goat were collected from a local abattoir and the corneas were detached carefully from the adhering tissues. It was immediately stored in normal saline (0.9% w/v) kept at 4°C, until used. Isolated cornea was held between the upper donor and lower receptor compartment of a Franz diffusion cell [28]. The cornea was firmly fixed to expose the epithelial surface (0.79 cm^2^) facing the donor compartment. The STF was used as receptor medium and the temperature of the receiver was set at 37 ± 0.1°C. Aliquots of samples (1 ml) were withdrawn at periodic time intervals and substituted with equivalent volume of new STF. The sample withdrawn from the receptor compartment was appropriately diluted and estimated for moxifloxacin by HPLC. The flux was determined as described in the literature [29].

### Differential scanning calorimetry (DSC)

Thermal behavior of moxifloxacin, physical mixture and MH7 were performed by DSC (DSC 60, Shimadzu, Kyoto, Japan). Samples were taken in crimped aluminium pan and sealed in airtight condition. The pan was scanned from 50–300°C at a uniform heating rate of 5°C/min. A blank aluminium pan was used as reference sample [30].

### Ocular irritation

Ocular irritation of MH7 was tested in albino rabbits (2–3 kg). The animals were housed under normal atmospheric conditions and allowed to move freely. The animals were acclimatized to the laboratory environments for one week prior to the start of experiment. All animals used for the study were given unrestricted access to both water and food. The experiments were carried out by strictly following the guidelines stated by the Committee for the Purpose of Control and Supervision of Experiments on Animals (CPCSEA), Ministry of Fisheries, Animal Husbandry and Dairying, India. The protocol approved by the Institutional Animal Ethics Committee (IPS/PCEU/FAC10-11/002) for animal care at Nirma University was followed during the experiment. *In vivo* ocular irritation experiment was carried out according to the guidelines based on Draize technique [31]. Single instillation of 60 μl was applied in left eye of individual rabbit whereas the right untreated eye is considered as control. The sterile formulation was tested two times a day for 21 days. The rabbits were checked frequently for signs of sensitivity reactions particularly redness, swelling, cloudiness, edema, haemorrhage, discharge and blindness [32].

### *In vivo* pharmacokinetics

The amount of moxifloxacin diffused into the aqueous humour of the rabbit eyes after ophthalmic administration was determined to compare the ocular bioavailability between MH7 and commercial moxifloxacin ophthalmic drops (0.5% w/v). *In vivo* pharmacokinetic investigations were performed in New Zealand Albino rabbits (2–3 kg) with two groups (n = 6). The protocol approved by the Institutional Animal Ethics Committee (IPS/PCEU/FAC10-11/002) for animal care at Nirma University was followed during the experiment. Single topical instillation (60 μl of 0.5% w/v drug) of MH7 was dropped in the lower cul-de-sac of one eye of individual rabbit in the first group while similar strength and volume of commercial eye drops was instilled into second group of rabbits. In each case, the untreated eye was considered as control. Both eyelids of all rabbits were lightly closed for 2 min to increase the contact of drug with the corneal membrane. Before aqueous humour withdrawal, individual animal was anaesthetized by intramuscular administration of xylazine and ketamine [33]. The samples (20 μl) of aqueous humour collected using 29-gauge insulin syringe needle were mixed with acetonitrile, and stored immediately at -80°C until further investigation. The samples were centrifuged (5000 rpm for 10 min) and the organic layer was assessed for drug content by HPLC.

### Stability

The stability of MH7 batch was assessed as per the latest ICH guidelines. Appropriate quantity of ophthalmic formulations placed in amber-coloured vials were stored for 6 months in a stability chamber at accelerated storage condition (40 ± 2°C/75% ± 5% relative humidity) [26]. At various time intervals, the samples were taken out and estimated for important physicochemical parameters including gelling capacity, pH, viscosity and *in vitro* drug release. The shelf life of the optimized formulation was computed using the classical Arrhenius plot [34].

### Statistical analysis

The statistical interpretation of experimental data was carried out by one-way ANOVA (SPSS 23, Chicago, IL, USA). The difference in values at *p* < 0.05 is considered statistically significant.

## Results and discussion

### Evaluation of gels

*In situ* gels (M1-M14) were prepared and assessed for different parameters and the observed data are summarized in Table 3. The visual observation of M1-M14 signifies that the formulations varied from clear solution to turbid with transparent (T) or less transparent (L) or opaque (O) in nature (Table 3). The pH of formulations ranged between 6.0–6.4 and was not influenced by the polymers studied in the current investigation. Similarly, the drug content of the *in situ* gels were also comparable and is relatively high (>95%).

**Table 3:** Physicochemical characteristics of preliminary *in situ* gel formulations.

Parameters	Batch code													
	M1	M2	M3	M4	M5	M6	M7	M8	M9	M10	M11	M12	M13	M14
**Transparency**	T	T	T	L	L	L	T	T	L	O	L	O	O	O
**Gelling capacity**	+	++	+	+	+++	+++	+++	+++	++	+++	++	+++	++	+++
**Viscosity (cP) at 1 rpm**	607 ± 72	837 ± 81	378 ± 56	452 ± 869	1140 ± 126	3700 ± 264	2175 ± 112	3050 ± 226	-	-	-	-	-	-
**Gel strength (g)**	92 ± 8	98 ± 12	79 ± 16	86 ± 13	134 ± 9	168 ± 16	152 ± 17	157 ± 14	-	-	-	-	-	-

T–Transparent; L–Less Transparent; O–Opaque.

+ No gelation and gels slowly dissolves; ++ gelation immediate and remains for a few hours; +++ gelation immediate and remains for an extended period.

### *In vitro* gelation

The visual observation of time taken for gel formation, gel remains for the time period and gel dissolves was done for M1-M14 *in situ* gels [13]. Formulations (M5-M8, M10, M12 and M14) demonstrated immediate gelation and also was stable for an extended period (Table 3). However, the formulations M9 to M14 were not considered for further characterization of physicochemical properties as they failed in clarity test.

### Rheology

**Fig 1:**
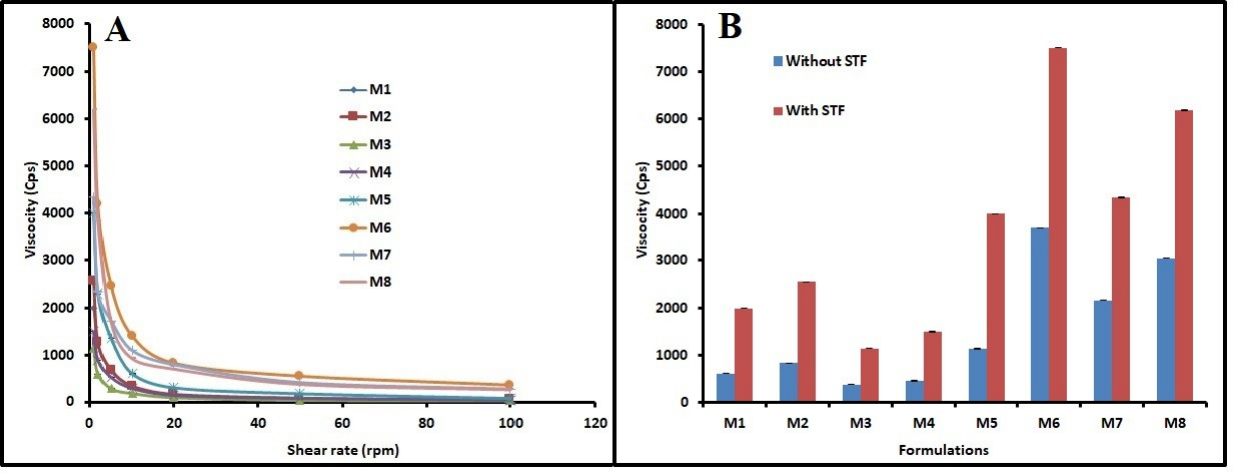
Rheology of *in situ* gels (M1–M8). (A) Rheological behavior and (B) viscosity with and without simulated tear fluid (STF).

The viscosity is a critical factor determining the ocular residence time of the instilled formulation [35]. The viscosity of M1-M8 are listed in Table 3. It was demonstrated that *in situ* gels (M1-M8) displayed pseudo-plastic flow or shear thinning rheological behavior as demonstrated by a drop in viscosity with higher angular velocity (Fig 1A). Viscosity of *in situ* gels (M1-M8) with and without STF is presented in Fig 1B. Percentage variation in viscosity after appropriate dilution of the formulations with STF is illustrated in S2 Fig. The data in the Figs 1B and S2 demonstrate that the dilution with STF remarkably improved the viscosity of M1-M8 gels formulated with gellan gum as well as sodium alginate. This is due to the fact that these polymers (gellan gum, sodium alginate and HPMC) have the inherent capacity to produce gel in companion with mono or divalent cations, available in STF analogous to the lachrymal fluid [5,13,36]. This peculiar phenomenon endorses the *in situ* gelling characteristic of the M1-M8 gels.

### Gel strength

Assessment of mucoadhesive force is an important parameter as it provides insight into the retention of formulation on the mucous membrane [37]. The gel strength as well as adhesive force of M1-M8 were determined using texture analyzer. It is apparent from the data in Table 3 that the gel strength varied considerably among the gels tested. The gel strength was relatively low when gel was prepared using single polymer (gellan gum or sodium alginate) (M1-M4). However, combining these two polymers drastically improved the gel strength (M5-M8), and the highest value (168 g) was noticed in M6, where in these polymers were combined with MC (viscosity enhancing agent). A similar trend was noticed with adhesive force as well and the values ranged between 3.7–5.8 N mm. However, M6 was less transparent as compared to gels prepared using HPMC (M7 and M8).

### Experimental design

**Fig 2:**
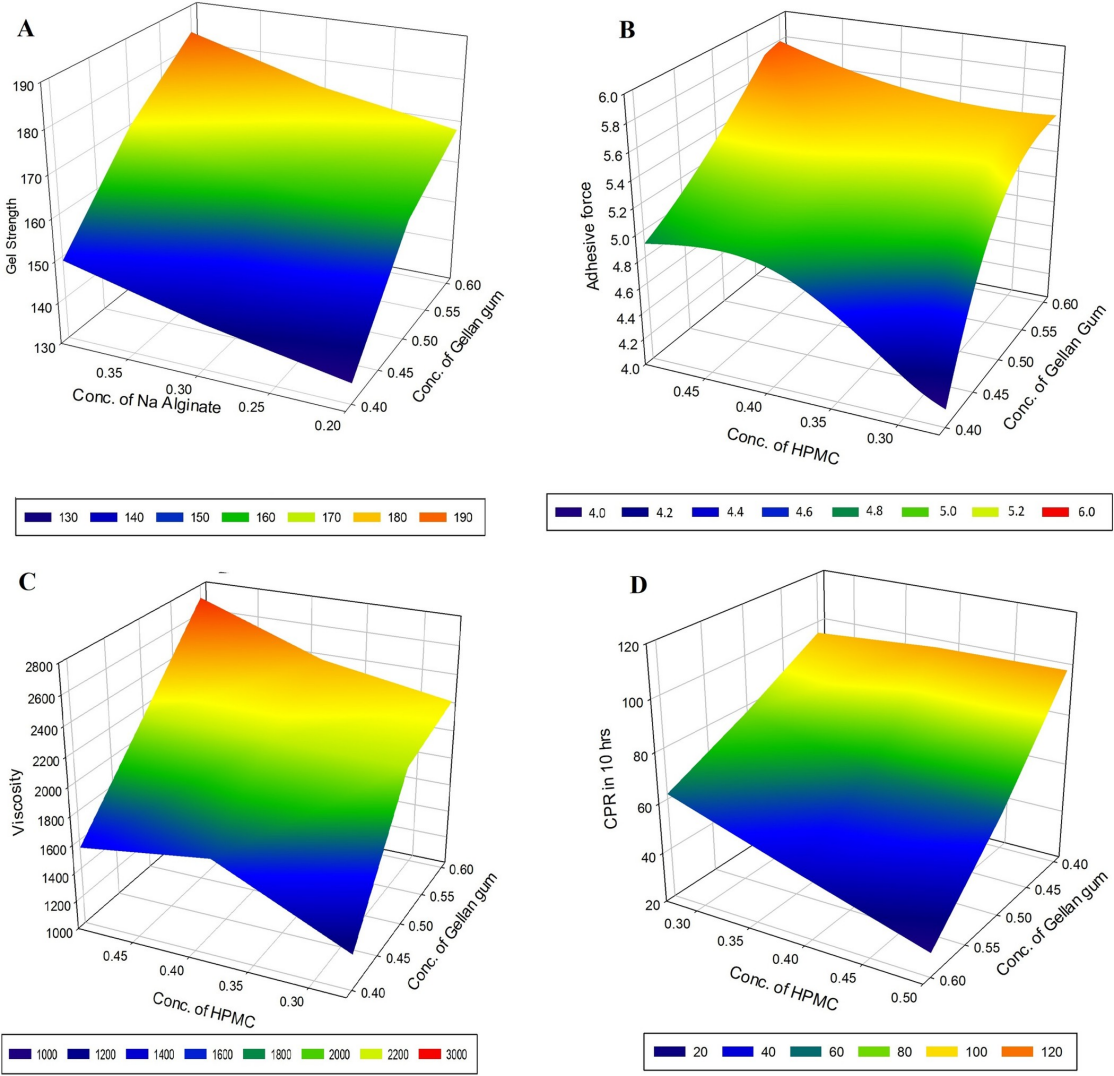
Response surface graphs representing (A) gel strength (B) adhesive force (C) viscosity and (D) release of drug in 10 h.

Data from the preliminary investigations of *in situ* gel formulation were used to select the polymers like gellan gum and sodium alginate whilst HPMC was opted as viscosity modifiers for further studies based on statistical design of experiments (DoE). Preparations composed of poloxamer 188 (M9-M10), poloxamer 407 (M11-M12), and carbopol 940P (M13-M14) were not transparent/clear and may interfere the vision, therefore, not included in design studies. Among the various formulations prepared with gellan gum, sodium alginate and HPMC, batches M7 and M8 exhibited satisfactory gel strength, viscosity and adhesive properties. Therefore, concentration of gellan gum, sodium alginate and HPMC was considered as independent variables denoted by X_1_, X_2_ and X_3_, respectively, and simplex lattice design was constructed to identify optimized gel. The design batches (MH1-MH7) were tested for gel strength, adhesive force, rheological property as viscosity and drug release after 10 h (Q_10_) (Table 4). The correlation between four dependent variables to the main effects (X_1_, X_2_ and X_3_) is shown in Response surface plots (Fig 2) and contour plots (S3 Fig). The data from MH1-MH7 batches of design were included for creating interpolated values with a software (Sigma plot; version 14.0).

**Table 4:** Designed batches coded, uncoded values and evaluation of experimental design batches.

Batch code	Variable levels			Actual values			Gel strength (g)	Adhesive force (N mm)	Viscosity (cP)	Q_10_ (%)
	X_1_	X_2_	X_3_	X_1_	X_2_	X_3_				
MH1	1	0	0	0.6	0.2	0.3	169 ± 14	5.5 ± 0.3	2260 ± 162	57.8 ± 5.2
MH2	0	1	0	0.4	0.4	0.3	149 ± 22	4.3 ± 0.8	1350 ± 121	95.4 ± 4.4
MH3	0	0	1	0.4	0.2	0.5	135 ± 18	4.9 ± 0.7	1546 ± 142	99.3 ± 3.6
MH4	0.5	0.5	0	0.5	0.3	0.3	164 ± 11	5.2 ± 0.8	2040 ± 184	70.5 ± 5.2
MH5	0.5	0	0.5	0.5	0.2	0.4	159 ± 21	5.3 ± 0.3	2150 ± 98	64.2 ± 6.1
MH6	0	0.5	0.5	0.4	0.3	0.4	141 ± 23	4.9 ± 0.3	1700 ± 94	99.1 ± 3.4
MH7	0.33	0.33	0.33	0.47	0.26	0.27	155 ± 18	5.1 ± 0.3	1950 ± 116	84.1 ± 4.2
MH8*	0.5	0.25	0.25	0.5	0.25	0.35	151 ± 16	5.2 ± 0.4	2180 ± 96	69.4 ± 5.7
MH9*	0.25	0.25	0.5	0.45	0.25	0.4	145 ± 17	5.1 ± 0.4	2010 ± 89	82.4 ± 6.2

Q_10_; cumulative percentage drug release after 10 h, * indicate check point batch.

### Effect of X_1_, X_2_ and X_3_ on gel strength

Interpretations from the statistical model indicates that all the three polymers considerably influence the gel strength. In addition, the increase in the amount of gelling agents also improved the gel strength of the formulations, probably due to polymer’s potential to improve the gelling power in presence of electrolytes present in STF [5,13,36]. Concentration of HPMC also affects gel strength but at lesser extent as compared to gellan gum and sodium alginate. Figs 2A and S3A shows significant influence of gellan gum, when compared to sodium alginate on gel strength.

### Effect of X_1_, X_2_ and X_3_ on adhesive force

The statistical analysis showed that increase in concentration of gellan, sodium alginate and HPMC polymers induced an improvement in adhesive force. These polymers either alone or in combinations enhanced adhesion with increase in concentration, except gellan gum-HPMC and gellan gum-sodium alginate-HPMC combinations. Figs 2B and S3B exhibits the positive influence of gellan gum and HPMC on adhesive force. Moreover, it is also observed that gellan gum have stronger influences on adhesive force (Figs 2B and S3B).

### Effect of X_1_, X_2_ and X_3_ on viscosity

The analysis also confirmed that increase in the amount of X_1_, X_2_, and X_3_ demonstrate a significant enhancement of viscosity. All the three polymers have the inherent viscosity building capacity promoted by the layer of solvent sheath surrounding the individual particle. The data also indicates that the increase in gellan gum and HPMC quantity proportionally enhanced the viscosity, as compared to alginate polymer. The response surface plot and contour plots depicted in Figs 2C and S3C displays the improvement in viscosity by gellan gum and HPMC. In addition, it is also proved that stronger influence of HPMC on viscosity.

### Effect of X_1_, X_2_ and X_3_ on drug release (Q_10_)

On evaluation of effect of three variables on drug release, it is observed that increase in amount of all three polymers can possibly decrease the release of the drug. From Figs 2D and S3D it was observed that increase in gellan gum and HPMC lead to more negative impact on the drug release. This could be explained by the fact that an increase in the gel strength and viscosity contributed by these polymers retarding the release rate of the drug.

The two check point batches (MH8* and MH9*) were examined for gel strength, adhesive force, rheological property (viscosity) and drug release studies and compared with predicted values observed in overlay plot of Design Expert software and found similarity between observed and predicted values (S1 Table).

### Drug release

**Fig 3:**
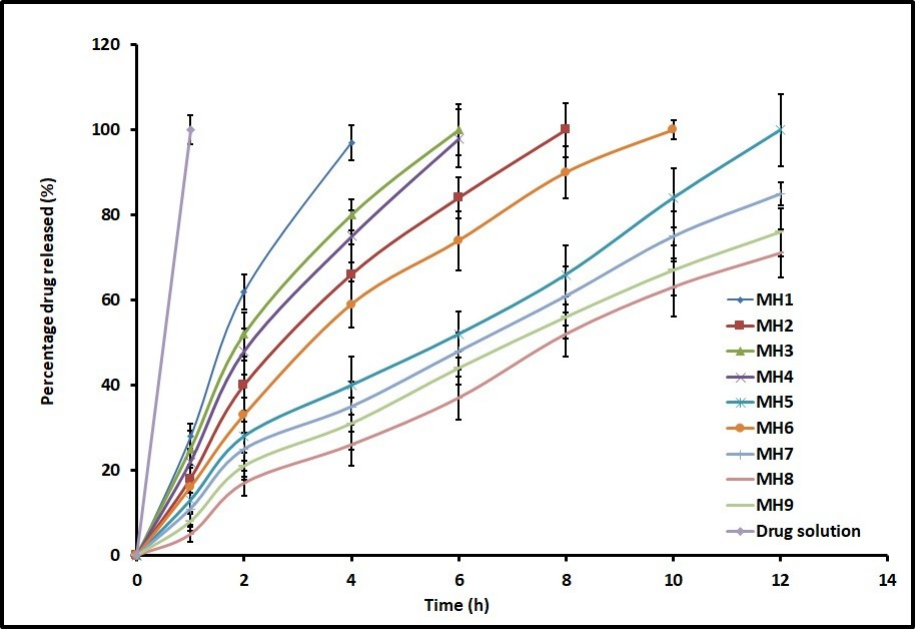
Comparison of percentage moxifloxacin release from *in situ* gels (M1–M8), check point batches (MH8 and MH9) and drug solution (control). The data represents average ± SD of six trials.

The drug release from *in situ* gels is imperative for absorption and to elicit therapeutic response. Comparison of the cumulative amount (%) of moxifloxacin released from MH1-MH7, check point batches (MH8 and MH9) and control is presented in Fig 3. It is apparent from the Fig 3 that the drug release profiles of MH1-MH7 *in situ* gels were relatively distinct, however, the percentage of moxifloxacin release increases as a function of time. To be specific, the drug release rate decreased marginally as follows; MH1>MH3>MH4>MH2>MH6> MH5> MH7. These minor variations in drug release could be correlated to the polymers used. For instance, the MH1-MH3 were fabricated using single polymer namely gellan gum, sodium alginate and HPMC, respectively, and hence demonstrated higher release rate. Similarly, MH4 (gellan gum and sodium alginate) and MH6 (sodium alginate and HPMC) also shown higher drug release, which contains equal amount of two polymers. However, the drug release rate was moderately low in MH5 and MH7. MH5 contains same level of gellan gum and HPMC, while MH7 contains all the three polymers in the same concentrations. Indeed, MH7 showed release profile comparable to the check point batches (MH8 and MH9) and was selected for further studies. On the other hand, the release of moxifloxacin in control experiments were immediate and exhibited almost complete release in 1 h. Overall, these data signify that release of moxifloxacin is influenced by the polymers studied.

Release rate data for MH7 was fitted using established mathematical models. The Sum of Square of Residuals (SSR) values that measure the deviation from the mean were found to be 80.55, 245.66, 239.11, 23.85, and 108.49 for Zero order, First order, Higuchi model, Korsmeyer-Peppas model, and Weibull model, respectively. Based on the data observed, moxifloxacin release from MH7 fit well to the Korsmeyer-Peppas expression (S2 Table), which is usually observed with gellan gum based *in situ* gels [38]. The observed n value (0.7837) suggests anomalous transport responsible for moxifloxacin release from MH7 [39,40].

A comparative *in vitro* release profile between MH7 and commercial eye drops of moxifloxacin is presented in S4 Fig. The data in S4 Fig signifies that the moxifloxacin release was considerably different at 15 min (17.78% and 3.72% for commercial eye drops and MH7, respectively) as well as 2 h (100% and 24.64% for commercial eye drops and MH7, respectively). The results demonstrated that the drug release from the MH7 was primarily controlled by the combination of polymers (gellan gum, sodium alginate and HPMC). Comparison of *in vitro* release between MH7 and commercial eye drops of moxifloxacin was accomplished by applying direct model-independent mathematical method, the similarity factor (F_2_). The calculated similarity factor value between MH7 and commercial eye drops was 16.94, proving that the both batches are dissimilar in terms of *in vitro* drug release profile according to the literature [41].

### *Ex vivo* permeation

**Fig 4:**
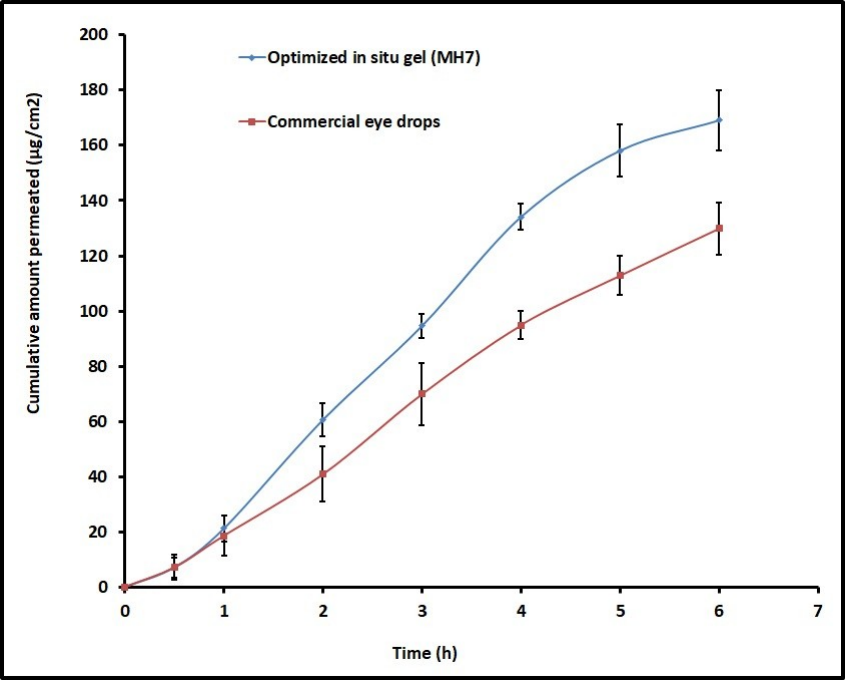
Comparison of moxifloxacin *ex vivo* permeation across the isolated goat cornea membrane from optimized *in situ* gel (MH7) and control (commercial eye drops). The data represents average ± SD of six trials.

Goat’s cornea was selected for the *ex vivo* drug permeation investigations for the design of experimental batches because it is multi-layered as well as simulate the condition of the human corneal membrane [22,42]. The cumulative amount of moxifloxacin penetrated via the cornea membrane from both MH7 and control are depicted in Fig 4. It is apparent from the Fig 4 that the drug release in the first hour was comparable in both cases. Subsequently, an increase in the drug permeation rate was recorded with MH7, in comparison to commercial eye drops. This finding is also in line with earlier reports wherein the *in situ* gels have significantly improved the trans-corneal permeation of various drugs [5,43,44]. The observed flux values (MH7; 31.02 μg/cm^2^/h and control; 22.78 μg/cm^2^/h) indicate significant difference (*p <* 0.005) in corneal permeation of moxifloxacin between MH7 and control. However, the lag time in both formulations were comparable (0.09 h). Overall, this study signifies that the moxifloxacin *in situ* gel formulation could significantly improve the corneal permeation.

### DSC

DSC technique was used to analyse the physical state of moxifloxacin including the transformation of thermodynamic properties that could have occurred inside the *in situ* gel. DSC thermograms of drug, physical mixture and *in situ* gel are depicted in S5 Fig. A sharp endothermic peak characterizing the melting point of moxifloxacin was observed at 262.87°C, demonstrating its crystalline character [45]. The diffraction pattern of physical mixture shown characteristic peaks of drug with reduced intensity at 262.87°C, and a broad endothermic peak at 94.17°C, which could be incurred as a result of melting point of polymers. The polymeric endotherm in the formulation MH7 shifted to 61.58°C, probably owing to *in situ* gel formation. However, the drug peak has disappeared in MH7 indicating the drug is in dissolved state in the *in situ* matrix gel system.

### Ocular irritation

Eye irritation score from individual rabbits was added to get the total irritation score that was subsequently divided by the total number of rabbits used for the ocular irritancy test to obtain the final eye irritation score. The calculated eye irritation score was 0.25 in control while for MH7 it was 0.57, which demonstrates good ocular tolerance like marketed formulation. Further, instillation of MH7 did not cause redness, swelling, or excessive lachrymation in the eye. Absence of ocular damage or unexpected clinical manifestations to the various eye regions (cornea, iris, or conjunctivae) were observed. Therefore, this study conclude that MH7 is safe and non-irritant for ocular administration.

### In vivo

**Fig 5:**
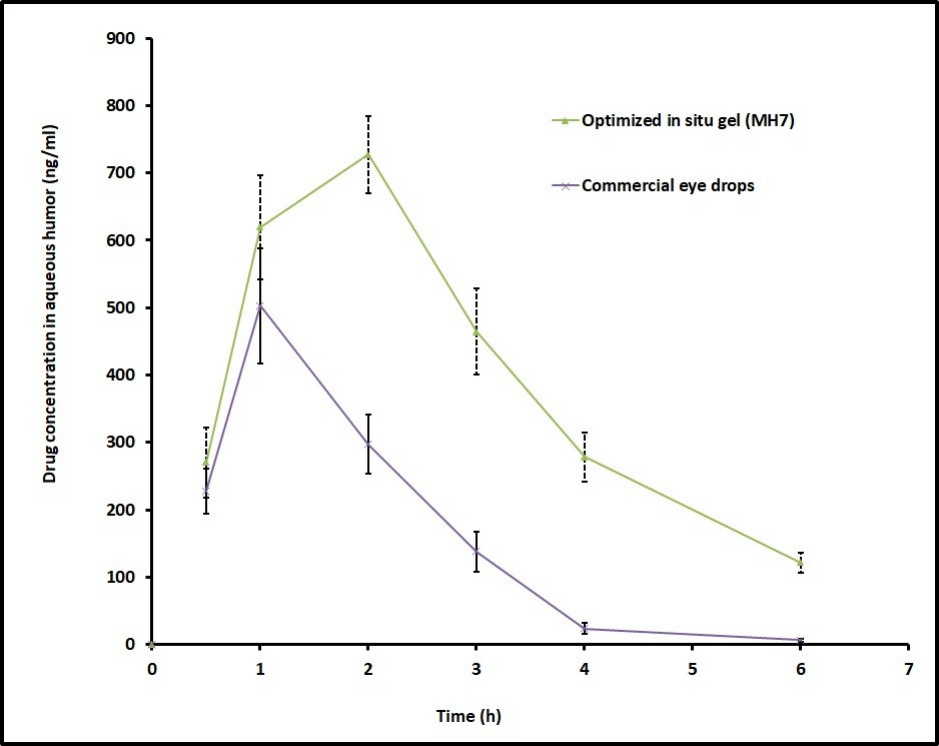
Comparison of mean moxifloxacin concentration in the aqueous humor following topical installation of optimized in situ gel (MH7) and control (commercial eye drops) in rabbits. The data represents average ± SD of six trials.

Ocular bioavailability is computed based on the amount of moxifloxacin permeated into the aqueous humour of rabbit eyes of first (MH7) and second groups (control). Various pharmacokinetic parameters including t_max_, C_max_ and AUC were computed from the graph plotted between concentrations (ng/ml) in aqueous humour and time (h) by non-compartment model analysis [46]. It is apparent from the Fig 5 that the pharmacokinetic parameters are markedly different for MH7 and control in aqueous humour in rabbits. After the completion of 1 h, the moxifloxacin level was elevated in the aqueous humour (619.31 ± 77.06 ng/ml and 503.09 ± 85.44 ng/ml in MH7 and control, respectively). However, the drug level was significantly higher (*p <* 0.0001) in MH7 at 2 h, but the moxifloxacin level declined sharply in conventional eye drops (Fig 5). These data signify that ophthalmic drops retained in the ocular cavity for short time because of extensive pre-corneal drug loss through nasolacrimal discharge and tear turn over. Further, the t_max_ value for MH7 was 2 h, while it was 1 h in control. On the other hand, MH7 showed higher C_max_ (727 ± 56 ng/ml) and greater AUC (2881 ± 108 ng h/ml) (*p <* 0.0001), when compared with commercial eye drops (C_max_; 503 ± 85 ng/ml and AUC; 978 ± 86 ng h/ml). Thus, it can be concluded from the available data that intraocular permeation of moxifloxacin was significantly improved by design and developing *in situ* gel system. This observation is also in agreement with *ex vivo* permeation data wherein the flux was significantly higher in MH7 (Fig 4). Therefore, ocular residence time of MH7 proves extended duration of action in comparison to commercial eye drops. The average drug concentration noticed in Fig 5 in the aqueous humour was more than the minimum effective concentration of moxifloxacin needed for therapeutic response for different pathogens causing eye infections [47].

### Stability

Stability data suggests no significant variation in gelling capacity, pH and viscosity of MH7 during storage. The *in vitro* drug release data after six-month stability cycles were evaluated by t-test considering two sample having equal variances. The observed *t* test value of 0.072 is well below the *t* critical value of 1.76, hence, demonstrates no statistically significant difference in MH7 after specified stability period. The decomposition of the drug was insignificant based on the calculated first-order degradation rate constant of MH7 (1.37 x 10^−4^ day^-1^). Since the long-term decomposition of moxifloxacin is <5%, an anticipated shelf life of 2 years can be estimated for MH7.

## Conclusions

In this research, experimental design using simplex lattice DoE was used to optimize the ion activated *in situ* gel contain moxifloxacin. *In situ* gel was successfully developed using a combination of gelling agents such as gellan gum and sodium alginate with HPMC. Including gellan gum with sodium alginate substantially decreased the amount of gellan gum and contribute additional adhesive and gelling strength to the formulations. The *in situ* gel extended drug release up to 12 h and was stable up to 6 months under accelerated stability conditions. This novel ophthalmic *in situ* gelling system is a feasible substitute to ophthalmic drops because of its inherent capacity to promote ocular bioavailability via sustained drug release, higher ocular permeability and prolonged residence time. Additional benefits including non-irritability, better miscibility with the lachrymal fluids, convenience of instillation, minimized frequency of application and total dose of moxifloxacin can lead to better patient compliance. Consequently, the developed topical *in situ* gel system (MH7) containing moxifloxacin possesses great potential for treating various ocular infections.

## Supporting information

S1 Fig QTS Texture analyzer used for measuring (a) gel strength and (b) adhesive force.
(DOCX)

S2 Fig Change in viscosity (%) of prepared in situ gels (M1-M8) by addition of simulated tear fluid (pH 7.4) STF (at 1.0 rpm). (DOCX)

S3 Fig Contour plots representing (A) gel strength (B) adhesive force (C) viscosity and (D) release of drug in 10 h.
(DOCX)

S4 Fig Comparison of percentage moxifloxacin release from MH7 and commercial moxifloxacin ophthalmic drops. (DOCX)

S5 Fig Differential scanning calorimetric curves of moxifloxacin, physical mixture and optimized *in situ* gel (MH7). (DOCX)

S6 Fig (JPG)

S1 Table Comparison of the observed value with predicted values of check point batches. (DOCX)

S2 Table Model fitting for selected in situ gel (MH7). (DOCX)
