# Supplementary figures and images for: Experimental design, formulation and in vivo evaluation of a novel topical in situ gel system to treat ocular infections
Source: PLoS One. 2021 Mar 19;16(3):e0248857. doi: 10.1371/journal.pone.0248857 (PMC7978349; doi:10.1371/journal.pone.0248857)

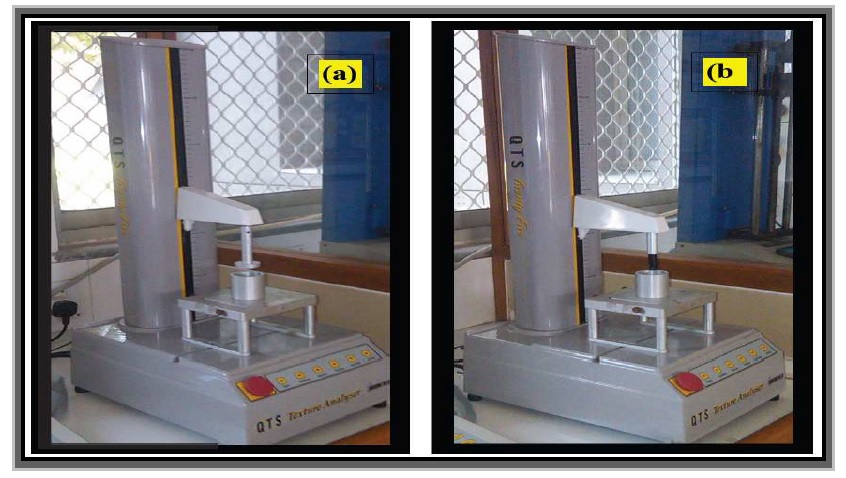


**S1 Fig.**QTS Texture analyzer used for measuring (a) gel strength and (b) adhesive force.

Supplement: S1 Fig — QTS Texture analyzer used for measuring (a) gel strength and (b) adhesive force. (DOCX) [file pone.0248857.s001.docx]

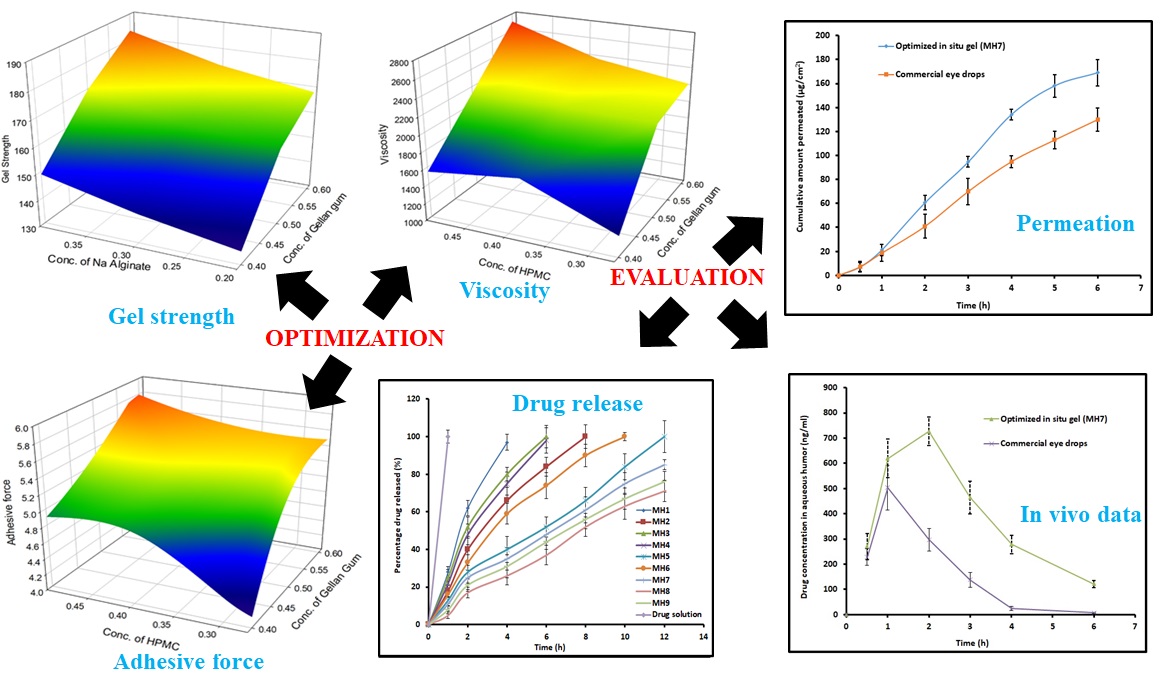

Supplement: S6 Fig — (JPG) [file pone.0248857.s006.jpg]
