# Supplementary material for: Experimental design, formulation and in vivo evaluation of a novel topical in situ gel system to treat ocular infections
Source: PLoS One. 2021 Mar 19;16(3):e0248857. doi: 10.1371/journal.pone.0248857 (PMC7978349; doi:10.1371/journal.pone.0248857)
Supplement: S2 Fig — (DOCX) [file pone.0248857.s002.docx]

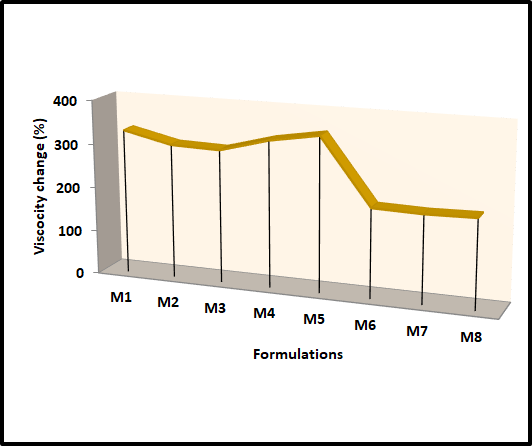


**S2 Fig.** Change in viscosity (%) of prepared in situ gels (M1-M8) by addition of simulated tear fluid (pH 7.4) at 1.0 rpm.
