# Supplementary material for: Experimental design, formulation and in vivo evaluation of a novel topical in situ gel system to treat ocular infections
Source: PLoS One. 2021 Mar 19;16(3):e0248857. doi: 10.1371/journal.pone.0248857 (PMC7978349; doi:10.1371/journal.pone.0248857)
Supplement: S3 Fig — Contour plots representing (A) gel strength (B) adhesive force (C) viscosity and (D) release of drug in 10 h. (DOCX) [file pone.0248857.s003.docx]

**
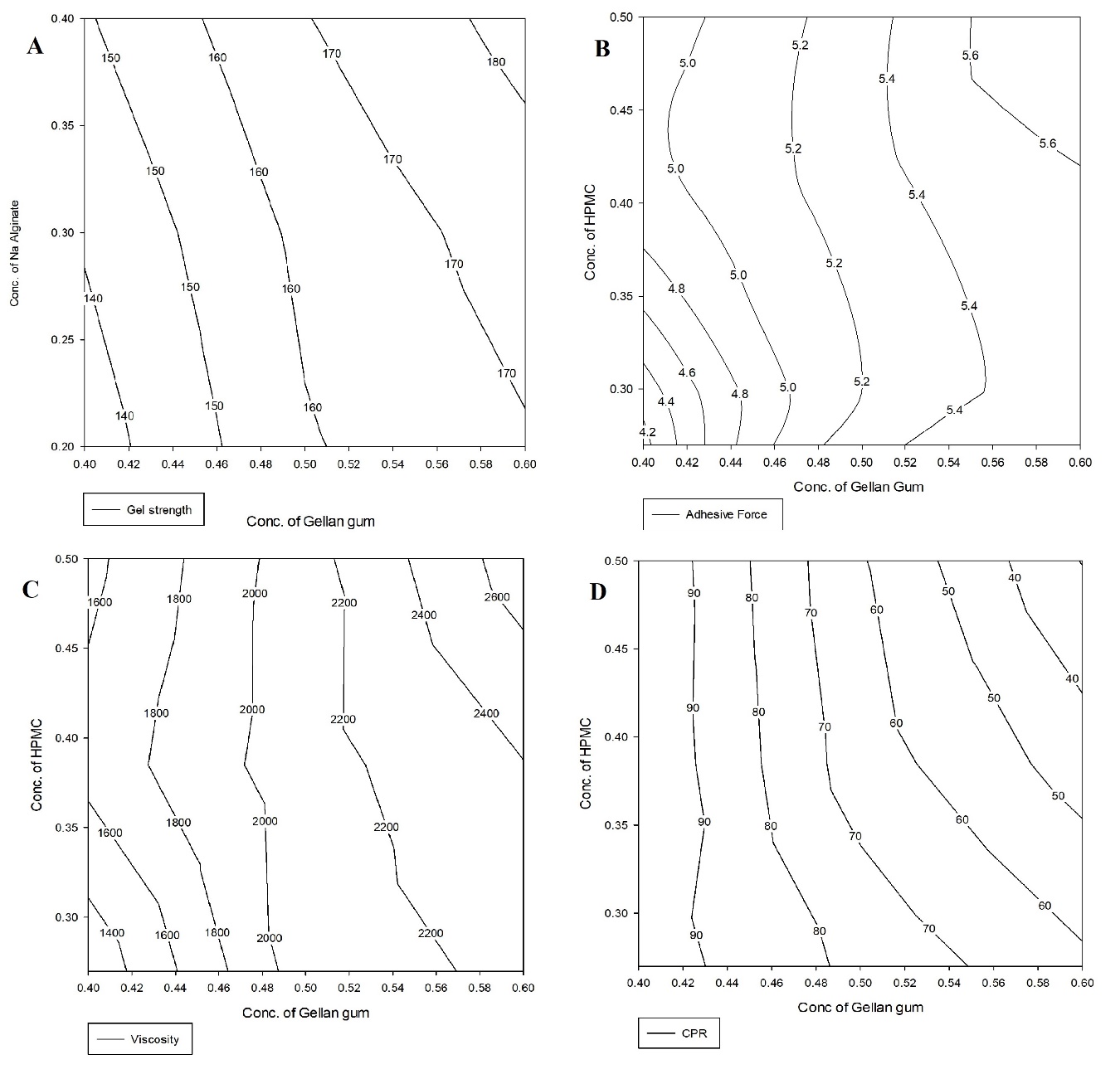
S3 Fig.** Contour plots representing (A) gel strength (B) adhesive force (C) viscosity and (D) release of drug in 10 h.
