# Supplementary material for: Experimental design, formulation and in vivo evaluation of a novel topical in situ gel system to treat ocular infections
Source: PLoS One. 2021 Mar 19;16(3):e0248857. doi: 10.1371/journal.pone.0248857 (PMC7978349; doi:10.1371/journal.pone.0248857)
Supplement: S5 Fig — (DOCX) [file pone.0248857.s005.docx]

**
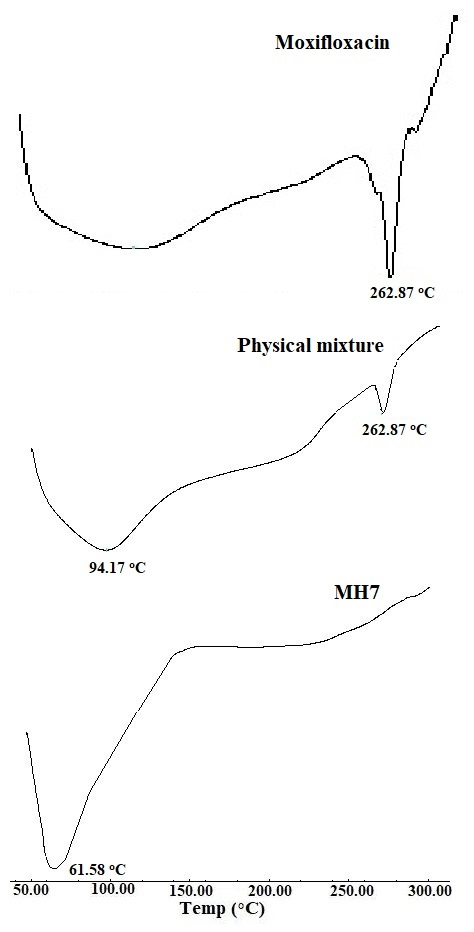
**

**S5 Fig.** Differential scanning calorimetric curves of moxifloxacin, physical mixture and optimized *in situ* gel (MH7).
