# Supplementary material for: Experimental design, formulation and in vivo evaluation of a novel topical in situ gel system to treat ocular infections
Source: PLoS One. 2021 Mar 19;16(3):e0248857. doi: 10.1371/journal.pone.0248857 (PMC7978349; doi:10.1371/journal.pone.0248857)
Supplement: S1 Table — (DOCX) [file pone.0248857.s007.docx]

**S1 Table.** Comparison of the observed value with predicted values of check point batches.

| **Batch code** | **Responses** | | | | | | | |
| --- | --- | --- | --- | --- | --- | --- | --- | --- |
|  | **Gel strength (g)** | | **Adhesive force (A)**  **(N mm)** | | **Viscosity (cp)** | | **Q10 (%)** | |
|  | **Observed** | **Predicted** | **Observed** | **Predicted** | **Observed** | **Predicted** | **Observed** | **Predicted** |
| **MH8*** | 151 | 154 | 5.2 | 5.0 | 2180 | 2153 | 69.4 | 67.6 |
| **MH9*** | 145 | 147 | 5.1 | 4.8 | 2010 | 1979 | 82.4 | 82.1 |

Q_10_; cumulative percentage drug release after 10 h, * indicate check point batch
