# Supplementary material for: Experimental design, formulation and in vivo evaluation of a novel topical in situ gel system to treat ocular infections
Source: PLoS One. 2021 Mar 19;16(3):e0248857. doi: 10.1371/journal.pone.0248857 (PMC7978349; doi:10.1371/journal.pone.0248857)
Supplement: S2 Table — (DOCX) [file pone.0248857.s008.docx]

**S2 Table.** Model fitting for selected in situ gel (MH7).

| **Model Name** | **Multiple R** | **r2** | **X variable** | **Slope** | **SSR** | **Fischer Ratio** |
| --- | --- | --- | --- | --- | --- | --- |
| **Zero order** | 0.9837 | 0.9874 | 6.8683 | 5.5826 | 80.5546 | 13.4258 |
| **First order** | 0.9815 | 0.9634 | -0.0642 | 2.0348 | 245.6589 | 40.9431 |
| **Higuchi** | 0.9811 | 0.9626 | 25.1766 | -8.8556 | 239.1120 | 39.8520 |
| **Korsmeyer – Peppas** | 0.9928 | 0.9917 | 0.7837 | -0.9144 | 23.8465 | 3.9744 |
| **Weibull Model** | 0.9890 | 0.9780 | 1.0498 | -0.9324 | 108.4911 | 18.0819 |
